# Impact of FGFR4 Gene Polymorphism on the Progression of Colorectal Cancer

**DOI:** 10.3390/diagnostics11060978

**Published:** 2021-05-28

**Authors:** Bei-Hao Shiu, Ming-Hong Hsieh, Wen-Chien Ting, Ming-Chih Chou, Lun-Ching Chang, Chi-Chou Huang, Shih-Chi Su, Shun-Fa Yang

**Affiliations:** 1 Institute of Medicine, Chung Shan Medical University, Taichung 402, Taiwan; shiubeihao@gmail.com (B.-H.S.); mhhpsy@hotmail.com (M.-H.H.); tingwenchien@gmail.com (W.-C.T.); cshy236@csh.org.tw (M.-C.C.); 2 Department of Surgery, Chung Shan Medical University Hospital, Taichung 402, Taiwan; 3 School of Medicine, Chung Shan Medical University, Taichung 402, Taiwan; 4 Department of Psychiatry, Chung Shan Medical University Hospital, Taichung 402, Taiwan; 5 Department of Mathematical Sciences, Florida Atlantic University, Boca Raton, FL 33431, USA; changl@fau.edu; 6 Whole-Genome Research Core Laboratory of Human Diseases, Chang Gung Memorial Hospital, Keelung 204, Taiwan; 7 Department of Dermatology, Drug Hypersensitivity Clinical and Research Center, Chang Gung Memorial Hospital, Linkou 333, Taiwan; 8 Department of Medical Research, Chung Shan Medical University Hospital, Taichung 402, Taiwan

**Keywords:** colorectal cancer, fibroblast growth factor receptor 4, single-nucleotide polymorphism, metastasis

## Abstract

Colorectal cancer (CRC) is a multifactorial malignancy, and its high incidence and mortality rate remain a global public health burden. Fibroblast growth factor receptor 4 (FGFR4) is a receptor tyrosine kinase that has been shown to play a key role in cancer development and prognosis via the activation of its downstream oncogenic signaling pathways. The present study aimed to explore the impact of *FGFR4* gene polymorphisms on the risk and progression of CRC. Three *FGFR4* single-nucleotide polymorphisms (SNPs), including rs1966265, rs351855, and rs7708357, were evaluated in 413 CRC cases and 413 gender- and age-matched cancer-free controls. We did not observe any significant association of three individual SNPs with the risk of CRC between the case and control group. However, while assessing the clinicopathological parameters, patients of rectal cancer possessing at least one minor allele of rs1966265 (AG and GG; AOR, 0.236; *p* = 0.046) or rs351855 (GA and AA; AOR, 0.191; *p* = 0.022) were found to develop less metastasis as compared to those who are homozygous for the major allele. Further analyses using the datasets from the Genotype-Tissue Expression (GTEx) Portal and The Cancer Genome Atlas (TCGA) revealed that rs351855 regulated FGFR4 expression in many human tissues, and increased FGFR4 levels were associated with the occurrence, advanced stage, and distal metastasis of colon adenocarcinoma. These data suggest that the amino acid change in combination with altered expression levels of FGFR4 due to genetic polymorphisms may affect CRC progression.

## 1. Introduction

Colorectal cancer (CRC) is among the most frequent malignancies worldwide [1]. In Taiwan, it is the most common cancer in males and the second in females, accounting for one of top common causes of cancer-related deaths [2]. Despite the current advance in surgery and other therapy options, the age-standardized mortality rate of CRC has increased over the years in Taiwan [3]. Such high incidence and death rates are largely attributed to the multifactorial nature of this neoplasm. It has been well documented that major external causes of CRC include but are not limited to diet and habitual exposure of cancer-causing substances, such as tobacco use and alcohol consumption [4]. Moreover, diverse genetic alterations that influence proteolysis, adhesion, angiogenesis, and cell growth have been demonstrated to mediate colorectal tumorigenesis [5]. In addition to host parameters, the dysbiosis of gut microflora lying at the intersection of those susceptibility factors mentioned above recently has emerged as a key determinant of CRC etiology [6]. Considering the high heterogeneity in CRC pathogenesis, all these disease risks appear to be mutually intertwined and necessary to assess the cancer prognosis.

Fibroblast growth factor receptor 4 (FGFR4) belongs to a highly conserved family of transmembrane receptor tyrosine kinases family, along with FGFR1-3. This family has been demonstrated to orchestrate a variety of oncogenic signaling involved in angiogenesis and epithelial–mesenchymal transition (EMT) in multiple types of cancers [7]. Similar with other members of the FGFR family, aberrant FGFR4 activation is linked to the formation of tumors, especially for those bearing *FGF19* amplification [8]. Converging observations indicate that dysregulation of FGFR4 downstream signaling pathways, such as Wnt/β-catenin [9], JAK/STAT [10], and PI3K-AKT [11], leads to enhanced cell growth and metastatic potential in cancer progression. Recently, an explosion of investigations has revealed the associations of *FGFR4* gene polymorphisms with the risk, prognosis, or treatment outcome of numerous cancer types, such as head and neck [12,13], lung [14], prostate [15,16,17], breast [18,19], colon [19], ovarian [20], liver [21], and uterine cervical cancer [22]. One *FGFR4* single-nucleotide polymorphism (SNP), rs351855, has been shown to promote tumorigenesis and cancer progression in a mouse model of breast cancer [23]. In addition to the oncogenic role, rs351855 may act as an expression quantitative trait locus (eQTL) as this allele was found to be associated with increased expression of FGFR4 protein [24]. Another *FGFR4* SNP, rs1966265, in combination with another *Klothoβ* gene variant were reported to link bile acid homeostasis to colonic transit. Nevertheless, the impact of *FGFR4* gene variants on the predisposition to CRC remains incompletely defined. Here, we performed a hypothesis-driven case-control study to assess the influences of *FGFR4* SNPs on the progression of CRC.

## 2. Materials and Methods

### 2.1. Subjects

This case-control study encompassed 413 patients with CRC and 413 cancer-free controls, with the approval by the institutional review board of Chung Shan Medical University Hospital in Taichung, Taiwan (IRB number CS1-20111). All participants, recruited from 2016 to 2020, provided informed written consent at enrollment. CRC patients were staged clinically at the time of diagnosis according to the TNM staging system of the American Joint Committee on Cancer (AJCC) [25]. Tumor differentiation was examined by a pathologist and rated according to the AJCC classification. In addition to the exclusion of history of cancer of any sites, subjects without self-reported asthma, diabetes, cardiovascular, neurological, and autoimmune diseases were enrolled in the control group. Demographic data on age and gender were recorded from each participant.

### 2.2. Genotyping

Genomic DNA was extracted from the whole blood using QIAamp DNA blood mini kits (Qiagen, Valencia, CA, USA). Evaluation of allelic discrimination for three FGFR4 SNPs (rs1966265, rs351855, and rs7708357) was conducted by using the TaqMan assay with an ABI StepOne™ Real-Time PCR System (Applied Biosystems, Foster City, CA, USA), and further evaluated with SDS version 3.0 software using the default settings (Applied Biosystems).

### 2.3. Statistical Analysis

The differences in demographic parameters between cases and cancer-free controls were estimated by using Fisher’s exact test. The adjusted odds ratios (AORs) with their 95% confidence intervals (CIs) for the association between genotype frequencies and the risk of CRC were calculated by multiple logistic regression models (dominant model and log-additive model) after controlling for potential confounders. Significant association between genotypes and FGFR4 expression levels in the GTEx portal was detected with the linear regression model and based on a *p*-value threshold determined by a web-based eQTL calculator on the GTEx portal. Tissue types (Colon and whole blood) were selected based on the location of cancer and tumor microenvironment. Clinical and mRNA expression data by RNA sequencing from patients with colon adenocarcinoma in The Cancer Genome Atlas (TCGA) were analyzed, and the differences of FGFR4 levels in the colon adenocarcinoma dataset were compared by Student’s *t*-test. Data were analyzed by using SAS statistical software (Version 9.1, 2005; SAS Institute Inc., Cary, NC, USA). A *p*-value < 0.05 was considered significant.

## 3. Results

### 3.1. Cohort Characteristics

In the present study, 413 CRC cases were recruited to explore the risk effect of FGFR4 gene polymorphisms on the development of colorectal neoplasm. Since age and gender are potential risk factors for CRC [26], 413 cancer-free controls with matched age and gender were enrolled to rule out potential confounders. The demographic and clinical characteristics of two study cohorts were evaluated (Table 1). Among the CRC cases, 106 and 307 suffered from cancers of the rectum and colon, respectively. Lymphatic spread and distal metastasis were observed in 48.4% and 16.7% of patients, respectively.

### 3.2. Association of FGFR4 Gene Polymorphism with the Progression of CRC

To examine the potential influence of FGFR4 gene polymorphisms on CRC progression, three SNPs from FGFR4 gene (rs1966265, rs351855, and rs7708357) were selected based on their wide associations with the development of various cancer types [13,14,16,27] and genotyped in this investigation. The distributions of genotype frequencies for each SNP in our cohort were evaluated (Table 2). We failed to individually observe any significant correlation of these FGFR4 variants with the occurrence of CRC between the case and control group. Further, we assessed the correlations of polymorphic genotypes of FGFR4 with clinicopathological characteristics of CRC patients. We found that patients of rectal cancer who carry at least one polymorphic allele of two missense SNPs (AG and GG for rs1966265; AOR, 0.236; 95% CI, 0.057–0.972; *p* = 0.046) (GA and AA for rs351855; AOR, 0.191; 95% CI, 0.046–0.786; *p* = 0.022) developed less distal metastasis (Table 3 and Table 4), yet the genetic effect was marginal as considering multiple testing. These data implicate a protective impact of FGFR4 gene polymorphisms on metastatic potential of cancers in the rectum.

### 3.3. Functional and Clinical Relevance of rs351855 in CRC

**Figure 1:**
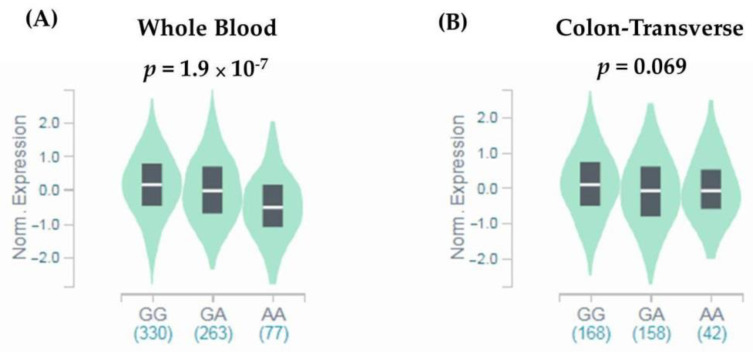
rs351855 regulates the expression of FGFR4. eQTL analysis of rs351855 in representative (**A**) whole blood and (**B**) colon tissues based on data from the GTEx portal. *p*-Values are calculated with the linear regression model.

**Figure 2:**
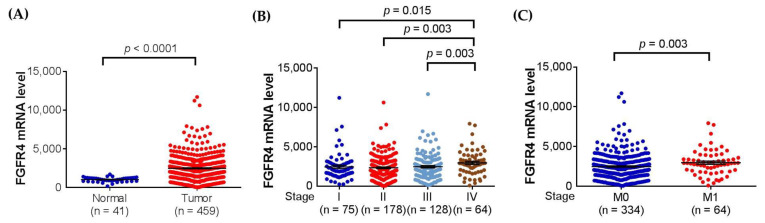
FGFR4 expression levels are associated with clinicopathological parameters in colon adenocarcinoma. Correlations of increased FGFR4 expression with the (**A**) occurrence, (**B**) advanced stage (stage IV), and (**C**) distal metastasis of colon adenocarcinoma from The Cancer Genome Atlas (TCGA) database. *p*-Values are calculated with Student’s *t* test and adjusted by using Bonferroni correction.

Since missense SNPs, rs1966265 and rs351855, were found to be associated with CRC metastasis, additional analyses using public datasets were performed to gain putative functional relevance of these two SNPs. We found that, in addition to the amino acid change, rs351855 regulated the expression of FGFR4 in multiple human tissues, such as whole blood (*p* = 1.9 × 10^−^^7^, based on a *p*-value threshold of 1.3 × 10^−4^) and, to a lesser extent, the colon (*p* = 0.069), as determined by eQTL studies (GTEx database) (Figure 1A,B). Moreover, further analysis of data from patients with colon adenocarcinoma in TCGA dataset revealed associations of increased FGFR4 expression levels with the occurrence, advanced stage, and distal metastasis of colon cancer (Figure 2A–C). There data support genetic associations detected in our study and suggest that the amino acid change in combination with altered expression levels of FGFR4 due to genetic polymorphisms may affect CRC progression.

## 4. Discussion

Accumulative evidence has manifested that CRC progression is a complex process orchestrated by both external and inherited factors. In the present study, we reported that *FGFR4* gene polymorphisms, rs1966265 and rs351855, mediated the metastatic potential of CRC but did not confer the susceptibility to colorectal malignancies. In addition, rs351855 may act as an eQTL for *FGFR4* gene expression, which correlated with the onset, advancement, and metastasis of colon cancer.

*FGFR4* SNP, rs351855, has been extensively reported for its association with the occurrence, progression, and prognosis of multiple tumor types [28]. This genetic variation (G > A) causes the substitution of glycine by arginine at position 388 in the transmembrane domain of the receptor [19]. Functional analysis of such amino acid change indicated that both polymorphic alleles are oncogenic [29]. Specifically, *FGFR4^gly388^* was the stronger inducer of tumor growth, whereas *FGFR4^arg388^* was the stronger inducer of cancer cell motility and migration. In addition to altered functionality, we found that rs351855 also regulated the expression levels of FGFR4 in many tissue types. Our data and findings from other investigations of solid tumors [30] have shown that elevated FGFR4 expression, leading to activation of downstream oncogenic signaling pathways, was highly associated with cancer development. In particular, we showed that the FGFR4 expression fluctuated with the genotypes of rs351855 in blood cells, implicating a differential impact of tumor-infiltrating immune cells on CRC progression according to distinct FGFR4 activation levels among patients of different rs351855 genotypes. Of note, unlike several previous reports linking rs351855 to cancer risk [15,17,19], we failed to detect a genetic association of rs351855 with the predisposition to CRC. This discrepancy may be, in part, accounted for by variations in the allele frequency of *FGFR4^arg388^* across the general population of different ethnic groups. It was estimated that *FGFR4^arg388^* has an allele frequency of ~13% in the African population, roughly 30% in the European and Northeast Asian populations, and nearly 45% in the Latino and Southeast Asian (such as Taiwan) populations [28,31]. As such, rs351855 was often demonstrated to be correlated with clinicopathological features of cancer but not with the occurrence of the disease.

In our study, another missense SNP, rs1966265, was found to be associated with the metastasis of CRC. This genetic polymorphism (A > G) leads to a valine to isoleucine change at position 10 in the signal peptide of the receptor. This *FGFR4* SNP in combination with another *Klothoβ* gene variant was previously shown to link bile acid homeostasis to colonic transit in irritable bowel syndrome (IBS) with diarrhea [32]. Exposure of cells of the gastrointestinal tract to repeated high physiologic levels of bile acids has been considered as a vital risk factor for gastrointestinal cancers [33]. In addition to host cells, bile acid malabsorption reshapes the composition of microflora in the ecosystem of the colon, and this bile acid-microbiota axis is believed to play a key role in intestinal carcinogenesis [34]. Other than gastrointestinal cancers, rs1966265 has been associated with the risk of developing breast cancer [35]. Here, we showed that patients with rectal cancer who possess at least one minor allele of rs1966265 developed less metastasis as compared with those who are homozygous for the major allele. This, for the first time, connects rs1966265 to the progression of rectal cancer.

Our data revealed an influence of *FGFR4* gene variations on the metastasis of rectal cancer; however, additional work is needed to address several limitations in the study. One is that the effects of *FGFR4* gene polymorphisms on the risk of developing CRC may be underestimated because of a lack of information regarding habitual drinking and smoking for adjustment. Another weakness is that the molecular mechanism underlying the protective role of rs1966265 in cancer metastasis remains an open question. Whether the genetic polymorphism (A > G) or substitution of valine by isoleucine controls the expression, membrane translocation, or downstream signaling of the receptor requires further investigations. Moreover, likely due to a limited genetic effect or sample size, the genetic relationship detected was marginal as considering multiple testing or in the absence of a replication cohort. In addition, the findings reported in this study may be unable to be extended to other ethnic groups unless replication experiments are performed.

## 5. Conclusions

In conclusion, our results demonstrate an association of *FGFR4* SNPs (rs1966265 and rs351855) with the metastatic potential in rectal cancer. Elevated FGFR4 expression levels contribute to the occurrence of colon cancer and an inclination to develop late-staged tumors and distant metastasis. These findings reveal a novel genetic relationship between *FGFR4* variants and CRC progression and suggest that the diseases of patients with specific genotypes may progress more aggressively than the others, thereby requiring extensive follow-up.

## Figures and Tables

**Table 1:** The distributions of demographical characteristics in 413 controls and 413 patients with CRC.

Variable	Controls (N = 413) *n* (%)	Patients (N = 413) *n* (%)	*p*-Value
Age (years)			
<65	219 (53.0%)	230 (55.7%)	0.442
≥65	194 (47.0%)	183 (44.3%)	
Gender			
Male	244 (59.1%)	259 (62.7%)	0.285
Female	169 (40.9%)	154 (37.3%)	
Tumor location			
Rectum		106 (25.7%)	
Left colon		181 (43.8%)	
Right colon		126 (30.5%)	
Stage			
I + II		203 (49.2%)	
III + IV		210 (50.8%)	
Tumor T status			
T1-T2		103 (24.9%)	
T3-T4		310 (75.1%)	
Lymph node status			
N0		213 (51.6%)	
N1 + N2		200 (48.4%)	
Metastasis			
M0		344 (83.3%)	
M1		69 (16.7%)	
Lymphovascular invasion			
No		263 (57.1%)	
Yes		177 (42.9%)	
Perineural invasion			
No		237 (57.4%)	
Yes		176 (42.6%)	
Pathologic grading			
Well		6 (1.5%)	
Moderately		375 (90.8%)	
Poorly		32 (7.7%)	

**Table 2:** Genotype distributions of FGFR4 gene polymorphisms in 413 controls and 413 patients with CRC.

Variable	Controls (N = 413) *n* (%)	Patients (N = 413) *n* (%)	OR (95% CI)	AOR (95% CI)
rs1966265				
AA	99 (24.0%)	122 (29.5%)	1.000 (reference)	1.000 (reference)
AG	212 (51.3%)	192 (46.5%)	0.735 (0.529–1.022)	0.730 (0.525–1.015)
GG	102 (24.7%)	99 (24.0%)	0.788 (0.537–1.155)	0.786 (0.535–1.154)
AG + GG	314 (76.0%)	291 (70.5%)	0.752 (0.552–1.025)	0.748 (0.548–1.020)
rs351855				
GG	124 (30.0%)	129 (31.2%)	1.000 (reference)	1.000 (reference)
GA	205 (49.6%)	202 (48.9%)	0.947 (0.692–1.296)	0.947 (0.692–1.297)
AA	84 (20.4%)	82 (19.9%)	0.938 (0.634–1.388)	0.935 (0.632–1.385)
GA + AA	289 (70.0%)	284 (68.8%)	0.945 (0.703–1.270)	0.944 (0.702–1.270)
rs7708357				
GG	402 (97.3%)	406 (98.3%)	1.000 (reference)	1.000 (reference)
GA	11 (2.7%)	7 (1.7%)	0.630 (0.242–1.642)	0.615 (0.236–1.605)
AA	0 (0.0%)	0 (0.0%)		
AG + AA	11 (2.7%)	7 (1.7%)	0.630 (0.242–1.642)	0.615 (0.236–1.605)

The odds ratio (OR) with their 95% confidence intervals were estimated by logistic regression models. The adjusted odds ratio (AOR) with their 95% confidence intervals were estimated by multiple logistic regression models after controlling for age and gender.

**Table 3:** Distribution frequency of the clinical status and FGFR4 rs1966265 genotype frequencies in 413 CRC patients.

Variable	All (N = 413)			Rectum (N = 106)			Colon (N = 307)		
	Stages			Stages			Stages		
	I + II	III + IV	*p*-Value	I + II	III + IV	*p*-Value	I + II	III + IV	*p*-Value
AA	58 (28.6%)	64 (30.5%)		12 (20.3%)	14 (29.8%)		46 (31.9%)	50 (30.7%)	
AG	98 (48.3%)	94 (44.8%)		32 (54.2%)	22 (46.8%)		66 (45.8%)	72 (44.2%)	
GG	47 (23.1%)	52 (24.7%)		15 (25.4%)	11 (23.4%)		32 (22.2%)	41 (25.2%)	
Dominant model			*p* = 0.188			*p* = 0.982			*p* = 0.179
Log-additive model			*p* = 0.967			*p* = 0.403			*p* = 0.619
	Tumor T status			Tumor T status			Tumor T status		
	T1 + T2	T3 + T4		T1 + T2	T3 + T4		T1 + T2	T3 + T4	
AA	32 (31.1%)	90 (29.0%)		8 (22.9%)	18 (25.4%)		24 (35.3%)	72 (30.1%)	
AG	45 (43.7%)	147 (47.4%)		17 (48.6%)	37 (52.1%)		28 (41.2%)	110 (46.0%)	
GG	26 (25.2%)	73 (23.5%)		10 (28.6%)	16 (22.5%)		16 (23.5%)	57 (23.8%)	
Dominant model			*p* = 0.692			*p* = 0.960			*p* = 0.588
Log-additive model			*p* = 0.967			*p* = 0.556			*p* = 0.589
	Lymph node status			Lymph node status			Lymph node status		
	Negative	Positive		Negative	Positive		Negative	Positive	
AA	64 (30.0%)	58 (29.0%)		13 (21.3%)	13 (28.9%)		51 (33.6%)	45 (29.0%)	
AG	100 (46.9%)	92 (46.0%)		31 (50.8%)	23 (51.1%)		69 (45.4%)	69 (44.5%)	
GG	49 (23.0%)	50 (25.0%)		17 (27.9%)	9 (20.0%)		32 (21.1%)	41 (26.5%)	
Dominant model			*p* = 0.189			*p* = 0.895			*p* = 0.147
Log-additive model			*p* = 0.672			*p* = 0.264			*p* = 0.240
	Metastasis			Metastasis			Metastasis		
	Negative	Positive		Negative	Positive		Negative	Positive	
AA	98 (28.5%)	24 (34.8%)		18 (20.2%)	8 (47.1%)		80 (31.4%)	16 (30.8%)	
AG	164 (47.7%)	28 (40.6%)		49 (55.1%)	5 (29.4%)		115 (45.1%)	23 (44.2%)	
GG	82 (23.8%)	17 (24.6%)		22 (24.7%)	4 (23.5%)		60 (23.5%)	13 (25.0%)	
Dominant model			*p* = 0.379			*p* = 0.046 ^a^			*p* = 0.984
Log-additive model			*p* = 0.568			*p* = 0.136			*p* = 0.854
	Lymphovascular invasion			Lymphovascular invasion			Lymphovascular invasion		
	Negative	Positive		Negative	Positive		Negative	Positive	
AA	71 (30.1%)	51 (28.8%)		17 (24.6%)	9 (24.3%)		54 (32.3%)	42 (30.0%)	
AG	110 (46.6%)	82 (46.3%)		34 (49.3%)	20 (54.1%)		76 (45.5%)	62 (44.3%)	
GG	55 (23.3%)	44 (24.9%)		18 (26.1%)	8 (21.6%)		37 (22.2%)	36 (25.7%)	
Dominant model			*p* = 0.969			*p* = 0.967			*p* = 0.936
Log-additive model			*p* = 0.697			*p* = 0.771			*p* = 0.486
	Perineural invasion			Perineural invasion			Perineural invasion		
	Negative	Positive		Negative	Positive		Negative	Positive	
AA	73 (30.8%)	49 (27.8%)		17 (24.3%)	9 (25.0%)		56 (33.5%)	42 (28.6%)	
AG	107 (45.1%)	85 (48.3%)		35 (50.0%)	19 (52.8%)		72 (43.1%)	62 (47.1%)	
GG	57 (24.1%)	42 (23.9%)		18 (25.7%)	8 (22.2%)		39 (23.4%)	36 (24.3%)	
Dominant model			*p* = 0.510			*p* = 0.485			*p* = 0.606
Log-additive model			*p* = 0.702			*p* = 0.770			*p* = 0.486
	Cell differentiation			Cell differentiation			Cell differentiation		
	Well/Moderately	Poorly		Well/Moderately	Poorly		Well/Moderately	Poorly	
AA	113 (29.7%)	9 (28.1%)		26 (24.8%)	0 (0.0%)		87 (31.5%)	9 (29.0%)	
AG	177 (46.5%)	15 (46.9%)		53 (50.5%)	1 (100.0%)		124 (44.9%)	14 (45.2%)	
GG	91 (23.9%)	8 (25.0%)		26 (24.8%)	0 (0.0%)		65 (23.6%)	8 (25.8%)	
Dominant model			*p* = 0.719						*p* = 0.742
Log-additive model			*p* = 0.844						*p* = 0.734

^a^ AOR (95% CI): 0.236 (0.057–0.972); The adjusted odds ratio (AOR) with their 95% confidence intervals were estimated by multiple logistic regression models after controlling for stages, tumor T status, lymph node status, metastasis, lymphovascular invasion, perineural invasion, cell differentiation.

**Table 4:** Distribution frequency of the clinical status and FGFR4 rs351855 genotype frequencies in 413 CRC patients.

Variable	All (N = 413)			Rectum (N = 106)			Colon (N = 307)		
	Stages			Stages			Stages		
	I + II	III + IV	*p*-Value	I + II	III + IV	*p*-Value	I + II	III + IV	*p*-Value
GG	62 (30.5%)	67 (31.9%)		13 (22.0%)	16 (34.0%)		49 (34.0%)	51 (31.3%)	
GA	101 (49.8%)	101 (48.1%)		34 (57.6%)	22 (46.8%)		67 (46.5%)	79 (48.5%)	
AA	40 (19.7%)	42 (20.0%)		12 (20.4%)	9 (19.2%)		28 (19.5%)	33 (20.2%)	
Dominant model			*p* = 0.276			*p* = 0.864			*p* = 0.249
Log-additive model			*p* = 0.878			*p* = 0.324			*p* = 0.664
	Tumor T status			Tumor T status			Tumor T status		
	T1 + T2	T3 + T4		T1 + T2	T3 + T4		T1 + T2	T3 + T4	
GG	34 (33.0%)	95 (30.6%)		9 (25.7%)	20 (28.2%)		25 (36.8%)	75 (31.4%)	
GA	47 (45.6%)	155 (50.0%)		18 (51.4%)	38 (53.5%)		29 (42.6%)	117 (49.0%)	
AA	22 (21.4%)	60 (19.4%)		8 (22.9%)	13 (18.3%)		14 (20.6%)	47 (19.7%)	
Dominant model			*p* = 0.627			*p* = 0.873			*p* = 0.619
Log-additive model			*p* = 0.964			*p* = 0.620			*p* = 0.649
	Lymph node status			Lymph node status			Lymph node status		
	Negative	Positive		Negative	Positive		Negative	Positive	
GG	68 (31.9%)	61 (30.5%)		14 (23.0%)	15 (33.3%)		54 (35.5%)	46 (29.7%)	
GA	103 (48.4%)	99 (49.5%)		33 (54.1%)	23 (51.1%)		70 (46.1%)	76 (49.0%)	
AA	42 (19.7%)	40 (20.0%)		14 (22.9%)	7 (15.6%)		28 (18.4%)	33 (21.3%)	
Dominant model			*p* = 0.237			*p* = 0.739			*p* = 0.153
Log-additive model			*p* = 0.806			*p* = 0.188			*p* = 0.285
	Metastasis			Metastasis			Metastasis		
	Negative	Positive		Negative	Positive		Negative	Positive	
GG	103 (29.9%)	26 (37.7%)		20 (22.5%)	9 (52.9%)		83 (32.5%)	17 (32.7%)	
GA	172 (50.0%)	30 (43.5%)		52 (58.4%)	4 (23.5%)		120 (47.1%)	26 (50.0%)	
AA	69 (20.1%)	13 (18.8%)		17 (19.1%)	4 (23.6%)		52 (20.4%)	9 (17.3%)	
Dominant model			*p* = 0.250			*p* = 0.022 ^a^			*p* = 0.824
Log-additive model			*p* = 0.337			*p* = 0.154			*p* = 0.766
	Lymphovascular invasion			Lymphovascular invasion			Lymphovascular invasion		
	Negative	Positive		Negative	Positive		Negative	Positive	
GG	75 (31.8%)	54 (30.5%)		19 (27.5%)	10 (27.0%)		56 (33.5%)	44 (31.4%)	
GA	114 (48.3%)	88 (49.7%)		35 (50.7%)	21 (56.8%)		79 (47.3%)	67 (47.9%)	
AA	47 (19.9%)	35 (19.8%)		15 (21.7%)	6 (16.2%)		32 (19.2%)	29 (20.7%)	
Dominant model			*p* = 0.882			*p* = 0.599			*p* = 0.866
Log-additive model			*p* = 0.872			*p* = 0.719			*p* = 0.654
	Perineural invasion			Perineural invasion			Perineural invasion		
	Negative	Positive		Negative	Positive		Negative	Positive	
GG	76 (32.1%)	53 (30.1%)		18 (25.7%)	11 (30.6%)		58 (34.7%)	42 (30.0%)	
GA	113 (47.7%)	89 (50.6%)		37 (52.9%)	19 (52.8%)		76 (45.5%)	70 (50.0%)	
AA	48 (20.3%)	34 (19.3%)		15 (21.4%)	6 (16.7%)		33 (19.8%)	28 (20.0%)	
Dominant model			*p* = 0.698			*p* = 0.717			*p* = 0.646
Log-additive model			*p* = 0.885			*p* = 0.494			*p* = 0.543
	Cell differentiation			Cell differentiation			Cell differentiation		
	Well/Moderately	Poorly		Well/Moderately	Poorly		Well/Moderately	Poorly	
GG	119 (31.2%)	10 (31.3%)		28 (26.7%)	1 (100.0%)		91 (33.0%)	9 (29.0%)	
GA	185 (48.6%)	17 (43.1%)		56 (53.3%)	0 (0.0%)		129 (46.7%)	17 (54.8%)	
AA	77 (20.2%)	5 (15.6%)		21 (20.0%)	0 (0.0%)		56 (20.3%)	8 (16.2%)	
Dominant model			*p* = 0.869			---			*p* = 0.626
Log-additive model			*p* = 0.723			---			*p* = 0.987

^a^ AOR (95% CI): 0.191 (0.046–0.786); The adjusted odds ratio (AOR) with their 95% confidence intervals were estimated by multiple logistic regression models after controlling for stages, tumor T status, lymph node status, metastasis, lymphovascular invasion, perineural invasion, cell differentiation.

## Data Availability

The data presented in this study are available on request from the corresponding author.
